# Construction and Validation of Novel Diagnostic and Prognostic DNA Methylation Signatures for Hepatocellular Carcinoma

**DOI:** 10.3389/fgene.2020.00906

**Published:** 2020-08-13

**Authors:** Ran Li, Liyan Shui, Junling Jia, Chao Wu

**Affiliations:** ^1^Life Sciences Institute, Zhejiang University, Hangzhou, China; ^2^State Key Laboratory for Diagnosis and Treatment of Infectious Diseases, National Clinical Research Center for Infectious Diseases, Collaborative Innovation Center for Diagnosis and Treatment of Infectious Diseases, The First Affiliated Hospital, College of Medicine, Zhejiang University, Hangzhou, China; ^3^Innovation Center for Precision Medicine, Zhongtong-Lanbo Diagnostic Ltd, Beijing, China

**Keywords:** hepatocellular carcinoma, DNA methylation, diagnostic signature, tumor risk, prognostic signature, cell cycle, eight gene panel

## Abstract

Hepatocellular carcinoma (HCC) is one of the most prevalent life-threatening human cancers and the leading cause of cancer-related mortality, with increased global incidence within the last decade. Identification of effective diagnostic and prognostic biomarkers would enable reliable risk stratification and efficient screening of high-risk patients, thereby facilitating clinical decision-making. Herein, we performed a comprehensive, robust DNA methylation analysis based on genome-wide DNA methylation profiling. We constructed a diagnostic signature with five DNA methylation markers, which precisely distinguished HCC patients from normal controls. Cox regression and LASSO analysis were applied to construct a prognostic signature with four DNA methylation markers. A one-to-one correlation analysis was carried out between genes of the whole genome and our prognostic signature. Exploration of the biological function and the role of the underlying significantly correlated genes was conducted. A mixed dataset of 463 HCC patients and 253 normal controls, derived from six independent datasets, was used to valid the diagnostic signature. Results showed a specificity of 96.84% and sensitivity of 96.77%. Class scores for the diagnostic signature were significantly different between normal controls, individuals with liver diseases, and HCC patients. The present signature has the potential to serve as a biomarker to monitor health in normal controls. Additionally, HCC patients were successfully separated into low-risk and high-risk groups by the prognostic signature, with a better prognosis for patients in the low-risk group. Kaplan-Meier and ROC analysis confirmed that the prognostic signature performed well. We found eight of the top ten genes to positively correlate with risk scores of the prognostic signature, and to be involved in cell cycle regulation. This eight-gene panel also served as a prognostic signature. The robust evidence presented in this study therefore demonstrates the effectiveness of the prognostic signature. In summary, we constructed diagnostic and prognostic signatures, which have potential for use in diagnosis, surveillance, and prognostic prediction for HCC patients. Eight genes that were significantly and positively correlated with the prognostic signature were strongly associated with cell cycle processes. Therefore, the prognostic signature can be used as a guide by which to measure responsiveness to cell-cycle-targeting agents.

## Introduction

Hepatocellular carcinoma (HCC) is a leading cause of cancer-related deaths worldwide, especially in developing countries, with more than half a million deaths per year (Yang and Roberts, 2010). Local ablation, surgical resection, and liver transplantation are recommended therapeutic options for early stage HCC (Llovet and Bruix, 2003; Forner et al., 2018). Based on current clinical practice guidelines, surgical resection is the optimal treatment for patients with a single tumor lesion and well-preserved liver function. However, even in this subgroup, the 5-year post-treatment recurrence rate approaches 70%, with no adjuvant therapy available (Villanueva et al., 2011; European Association for the Study of the Liver, 2018). Because of the highly heterogeneous nature of HCC, accurate diagnosis, which is mainly based on histological subtype and other markers associated with histology and immunohistochemistry, is crucial for choosing the proper treatment (Vogel et al., 2019). Pre-treatment evaluation of patients can help identify individuals with a high risk for recurrence and metastasis as well as poor prognosis. Such evaluation guides therapeutic strategies. Therefore, there is an urgent need to identify accurate and effective biomarkers for diagnosis and prognosis.

DNA methylation is a key epigenetic regulatory factor that typically results in gene silencing, playing an important role in cancer initiation and progression (Hattori and Ushijima, 2016; Koch et al., 2018). Numerous studies have shown that promoter hypermethylation is responsible for silencing of cancer tumor suppressor genes (Thienpont et al., 2016; Ma et al., 2017). Hypomethylation of oncogene promoters has also been observed in multiple types of cancer (Irizarry et al., 2009; Wang et al., 2018). Cancer-related DNA methylation is often detected earlier than the actual neoplastic transformation, with changes in DNA methylation correlated with early carcinogenesis (even prior to tumor formation), distant metastasis, and therapeutic sensitivity (Teschendorff and Widschwendter, 2012; Teschendorff et al., 2012; Zhuang et al., 2012; Hughes et al., 2013; Flanagan et al., 2017). Therefore, DNA methylation is a promising prognostic and diagnostic biomarker. Although a lot of novel biomarkers for HCC have been recently identified, there are few validated methylation biomarkers available for HCC (Qiu et al., 2017; Xu et al., 2017; Li et al., 2019). Owing to the lack of an unbiased comprehensive and systematic approach to whole-genome methylation analysis, many studies have overlooked the potential biological mechanisms associated with individual biomarkers.

The aim of this study was to identify and validate HCC DNA methylation biomarkers for diagnosis and prognosis. We analyzed whole-genome methylation profiles of HCC patients and normal controls from multicenter databases and employed multiple statistical methods to construct diagnostic and prognostic signatures. A diagnostic signature was validated in other independent datasets and a prognostic signature was combined with transcriptome datasets to explore the biological mechanisms underlying the signature. The results demonstrate diagnostic and prognostic signatures to be reliable and accurate for the diagnosis, surveillance, and prognosis of HCC patients.

## Materials and Methods

### Data Sources

In this study, whole-genome DNA methylation profiles based on the Illumina HumanMethylation450 BeadChip Assay data for 836 HCC patients and 303 normal controls were collected. Of these, profiles of 371 HCC patients and 50 normal controls with clinical survival information were obtained from The Cancer Genome Atlas (TCGA) using UCSC Xena^1^, with the remaining profiles of 461 HCC patients and 253 normal controls from six independent datasets downloaded from the Gene Expression Omnibus (GEO) database (GSE54503, GSE56588, GSE60753, GSE75041, GSE77269, and GSE89852). Gene expression profiles and clinical information of 371 HCC patients and 50 normal controls were downloaded from TCGA using UCSC Xena, with gene expression normalized using the upper quartile normalization method. Gene symbols corresponding to ensemble IDs were converted using gencode.v22.annotation.gene.probeMap^2^. Gene expression profiles based on the Illumina HiSeq RNA-Seq platform and clinical information of 232 Japanese HCC patients were collected from the International Cancer Genomics Consortium (ICGC, LIRI-JP) (International Cancer Genome Consortium, 2010). Gene expression matrix files based on Affymetrix microarray profiling and corresponding clinical information of 242 HCC patients were download from the GEO database (GSE14520). Most HCC and normal control samples were fresh-frozen clinical tissues, only two HCC samples were formalin-fixed paraffin-embedded (FFPE) clinical tissues. Details for these datasets are listed in Supplementary Table S3.

### Data Analysis

#### Genome-Wide DNA Methylation Preprocessing

To minimize the impact of certain low-quality and unreliable methylation probes in the TCGA dataset, two-step filtering criteria were adopted. In the first step, probes located on sex chromosomes or not mapping uniquely to the genome were removed. In the second step, only probes available in at least 90% of samples were considered. At each CpG site, methylation is quantified by the beta value (β-value) as β = M/(M+U), where M and U denote the methylated and unmethylated intensities, respectively (Du et al., 2010). Therefore, we can use β-values (ranging from 0 to 1) to estimate methylation levels of each CpG site. Missing β-values were estimated with the median β-value of the identical methylation marker of the remaining samples. After the above preprocessing steps, 370,011 of 485,512 methylation markers were retained. To achieve similar distribution of methylation β-values across patients, β-values were normalized using the function “normalizeBetweenArrays” with default parameters from the “limma” R package (Ritchie et al., 2015).

### Differential DNA Methylation Analysis

We randomly selected two-thirds of the 371 HCC samples from the TCGA dataset and all 50 normal controls of the TCGA dataset. For each marker, the mean quotient was calculated between average methylation β-value in HCC patients (Ave_*T*_) and normal controls (Ave_*N*_):

(1) $$Log_{2}\left(\mathrm{fold}\mathrm{change}\right)=Log_{2}\left[\frac{\text{max}\left(Ave_{T},0.01\right)}{\text{max}\left(Ave_{N},0.01\right)}\right]$$

We calculated statistical significance using the multiple testing-corrected Wilcoxon test (FDR). The standard deviation (SD) of the β-value of each marker was also calculated (SD_*T*_: SD of β-value in HCC patients; SD_*N*_ : SD of β-value in normal controls).

### Construction of a Diagnostic Signature

The preselection of methylation markers for diagnostic analysis was based on the following three criteria: first, Ave_*T*_ > 0.3 or Ave_*N*_ > 0.3; second, |Log_2_ (fold change)| > 1 and FDR < 0.01; third, SD_*T*_ < 0.2 and SD_*N*_ < 0.2. After preselection, 1306 candidate markers were available for further analysis. For each marker, the highest balanced accuracy (BA) for optimal β-value threshold was obtained as follows (Chang and Chen, 2019; Gao et al., 2019):

(2) $$\mathrm{Balanced}\mathrm{accuracy}=\left(\frac{TP}{TP+FN}+\frac{TN}{TN+FP}\right)/2$$

Where TP is the number of HCC patients correctly identified as HCC; FP is the number of normal controls incorrectly identified as HCC; *FN* is the number of HCC patients incorrectly identified as normal; TN is the number of normal controls correctly identified as normal. We ranked candidate markers based on BA (from high to low). Then we built a diagnostic signature with the following steps:

1. We built a matrix whose column is the number of simulations and the row is the top methylation markers (Supplementary Figure S2A).
2. For the first column as an example, we sampled 186 HCC patients and 25 normal controls from the TCGA dataset. We applied these to the diagnosis model as an increase in methylation markers, to calculate BA individually as follows:
  (3) $$Sum_{ij}=\left\{ \begin{array}{c} Sum_{\left(i-1\right)j}+\beta_{ij},Ave_{T}>Ave_{N}\\ Sum_{\left(i-1\right)j}-\beta_{ij},Ave_{T}\leq Ave_{N} \end{array}\right.$$
  Where β_**ij**_ is the β-value of marker *i* in sample *j*, Sum_*ij*_ is the sum of β-values of the top *i* markers in sample j.
  (4) MT=Q1-1.5(Q3-Q1)
  Here, we assumed that average Sum_*ij*_ in HCCs greater than average Sum_*ij*_ in normal controls. Q*_*i*_* is the lower quartile of Sum_*ij*_ in HCCs, Q3 is the upper quartile of Sum_*ij*_ in HCCs. Individuals with Sum_*ij*_ not less than MT were identified as HCC, otherwise individuals were identified as normal. Then, equation 2 was used to calculate balanced accuracy.
3. To avoid dataset bias, we repeated step 2. For the top methylation markers, we got the average BA with increase of simulation number (Supplementary Figure S1).

### Construction of a Predictive Signature for Prognosis and Survival

We randomly divided the TCGA dataset into two cohorts. The first two-thirds were used as a training dataset for construction of a prognostic signature. The remaining one-third were used as a validation dataset for verification of the performance of the prognostic signature. Methylation markers with Ave_*T*_ > 0.2 for the training dataset were selected. Then, we evaluated whether each marker was able to distinguish between high-risk and low-risk HCC patients using the training dataset based on the following:

1. Median methylation β-value in HCCs (MM).
2. Average survival time of HCCs with methylation β-value not less than MM and dead status (Time_*H*_).
3. Average survival time of HCCs with methylation β-value less than MM and dead status (Time_*L*_).
4. Fold change of average survival time $\text{FC}=\frac{{\text{Time}}_{H}}{{\text{Time}}_{L}}$.

Markers with *FC* greater than 2 or less than 0.5 were used to perform univariate Cox proportional hazard regression analysis. Markers that significantly (*p* < 0.01) correlated with overall survival (OS) of HCC patients were identified as candidate markers. Subsequently, we applied a variable selection method that is suitable for high-dimensional data: Least Absolute Shrinkage and Selection Operator (LASSO) (Tibshirani, 1997; Zhang and Lu, 2007). We adopted 100-fold cross-validation to select the optimal lambda value to minimize the prediction error, known as “min” lambda. A total of 22 candidate markers were selected by the LASSO-Cox method and incorporated into the multivariate Cox regression analysis. Finally, an optimal prognostic signature consisting of four DNA methylation markers was constructed (Supplementary Figure S1). We obtained a combined risk-score by multiplying coefficient estimates and methylation β-values of the marker matrix in the training and validation datasets.

To determine the predictability of the prognostic signature, Kaplan-Meier estimator and log-rank test were performed. In this manner, we investigated whether HCC patients could be divided into low-risk and high-risk groups using a median risk-score obtained from the training dataset. To evaluate the specificity and sensitivity of the prognostic signature for survival prediction, the area under ROC curve (AUC) was calculated by time-dependent ROC analysis. All the above analysis was conducted in R version 3.6.0 with the following packages: “limma,” “glmnet,” “timeROC,” “survival,” “survminer,” “ggplot2,” “forestplot,” “pheatmap,” “FactoMineR,” and “factoextra.”

## Results

### Constructing and Validating a Diagnostic Signature for HCC Based on DNA Methylation

**FIGURE 1:**
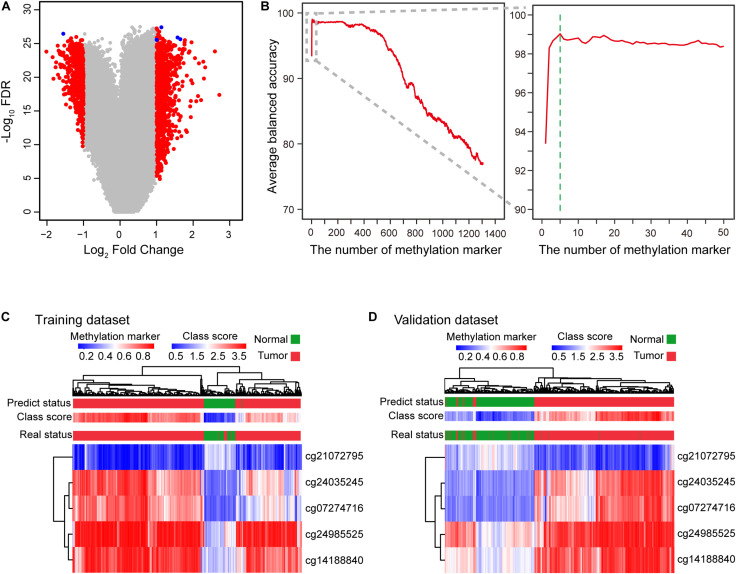
Construction of the diagnostic signature consisting of five DNA methylation markers. **(A)** Five DNA methylation markers were selected to construct the diagnostic signature. Volcano plot showing the biological significance (log_2_ fold change (FC)) on the *X* axis and the corresponding statistical significance (−log_10_ false discovery rate (FDR)) on the *Y* axis of markers between HCC patients and normal controls. A total of 1306 candidate markers (red and blue) were initially selected, with five markers (blue) finally selected to construct the diagnostic signature. **(B)** Curve of average balanced accuracy with increasing number of methylation markers. The green dotted line indicates that the diagnostic signature with five DNA methylation markers has the highest average balanced accuracy for sampling 100 times. **(C,D)** Heatmap of five DNA methylation markers selected to construct the diagnostic signature for the training (TCGA) **(C)** and validation **(D)** datasets (GSE54503, GSE56588, GSE60753, GSE75041, GSE77269, and GSE89852).

We first evaluated genome-wide DNA methylation profiles of the TCGA dataset. Eligible methylation markers had a significantly differential β-value for HCCs and normal controls, small β-value standard deviation (SD<0.2) for both HCCs and normal controls, and an average methylation β-value greater than 0.3 for either HCCs or normal controls. These three stringent criteria were defined for the initial screening of candidate methylation markers (see section “Materials and Methods”). After the prescreening process, 1306 methylation markers able to distinguish and identify HCCs and normal controls were selected (Figure 1A and Supplementary Figure S1). We ranked these candidate markers based on BA. Two-thirds of methylation profiles of the HCC patients and normal controls from the TCGA dataset were sampled and applied to the diagnostic model to assess an increase in methylation markers. To avoid bias of the sampling dataset, we simulated the process for sampling 10, 50, 100, 300, 500, 1000 times, respectively, to calculate the average BA (Figure 1B and Supplementary Figure S2A, see section “Materials and Methods”). After 100 simulations, the diagnostic signature consisting of the top 5 methylation markers (cg24985525, cg24035245, cg21072795, cg07274716, and cg14188840) discriminated HCC patients and normal controls with the highest average BA (Figure 1B and Supplementary Figure S2B, Supplementary Tables S1, S2). These five DNA methylation markers correspond to LDL receptor-related protein 5 (*LRP5*), T-box transcription factor 15 (*TBX15*), NCK associated protein 1 like (*NCKAP1L*), paired like homeodomain 1 (*PITX1*), and homeobox A10 (*HOXA10*), respectively. The use of too few or too many markers for the diagnosis signature can decrease BA and increase the instability of BA for different datasets (Figure 1B and Supplementary Figure S2B, Supplementary Table S1). Therefore, we constructed a diagnostic prediction signature with five DNA methylation markers created by the class-score formula as follows: class-score = β-value of cg24985525 + β-value of cg24035245 + β-value of cg21072795 + β-value of cg07274716 + β-value of cg14188840. The class-score (1.585) of the training dataset was the cutoff point. Samples with class-scores less than 1.585 were diagnosed as normal, while samples greater than or equal to the cutoff point were diagnosed as HCC or risk for HCC.

**FIGURE 2:**
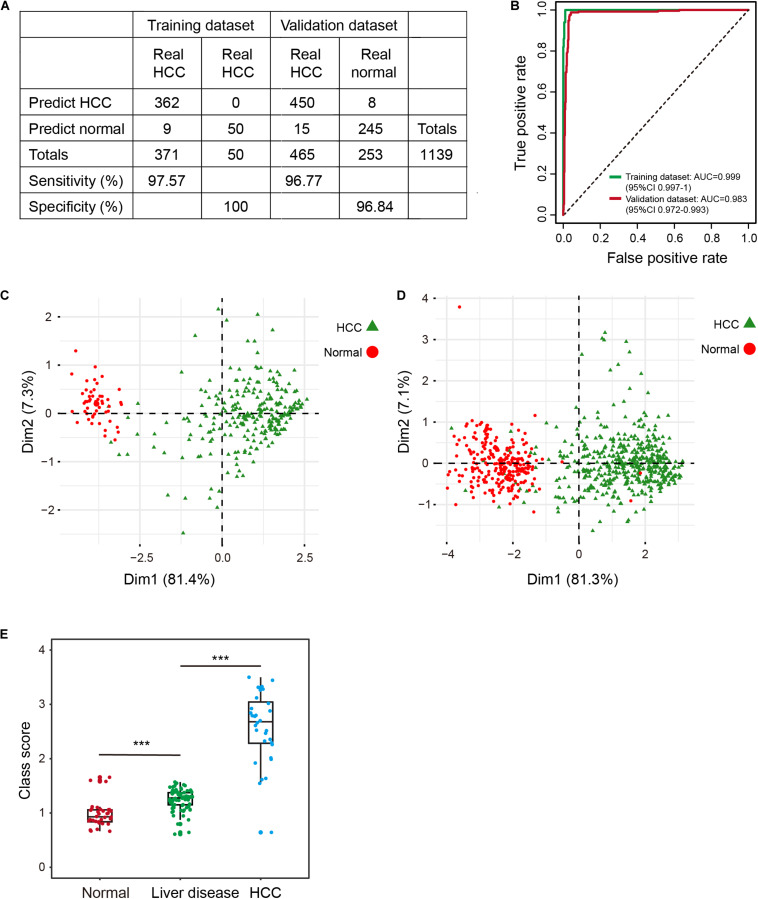
Evaluation of the diagnostic signature in the training (TCGA) and validation datasets (GSE54503, GSE56588, GSE60753, GSE75041, GSE77269, and GSE89852). **(A)** The predicted results of the diagnostic signature in the training and validation datasets. **(B)** ROC curves of the diagnostic signature in the training and validation datasets. **(C,D)** PCA analysis of the five DNA methylation markers between HCC patients (green) and normal controls (red) in the training **(C)** and validation **(D)** datasets. ***p < 0.001 from Wilcoxon test. **(E)** Distribution of class-scores for the diagnostic signature of normal controls, individuals with liver diseases (alcoholism and HBV/HCV cirrhosis), and HCC patients in GSE60753 dataset.

To profile the distribution of the β-values of the five DNA methylation markers of the training and validation datasets, unsupervised hierarchical clustering analysis was performed. The heatmap showed that the β-value of the five markers of the diagnostic signature were significantly different between HCCs and normal controls. Higher methylation β-values for HCCs were obtained for cg24985525, cg24035245, cg07274716, and cg14188840. In contrast, methylation levels of cg21072795 were relatively low for HCCs (Figures 1C,D). However, the use of the five DNA methylation markers enabled HCCs to be distinguished from normal controls. In addition, the diagnostic signature based on the five DNA methylation markers had better diagnostic performance (Figures 1C,D). Applying the diagnostic signature, we obtained a specificity of 100% and sensitivity of 97.57% in the training dataset (Figure 2A). To substantiate the stability and accuracy of the diagnostic signature, validation analysis was performed in an external dataset consisting of six independent datasets (GSE54503, GSE56588, GSE60753, GSE75041, GSE77269, and GSE89852) (Supplementary Table S3). The results showed the diagnostic signature to maintain its discriminative power, yielding a sensitivity of 96.77% and specificity of 96.84% (Figure 2A). To investigate the specificity of the diagnostic signature for diagnostic analysis, the AUC values of the receiver operating characteristic (ROC) curves were calculated by time-dependent ROC analysis. The results demonstrated the diagnostic signature could differentiate HCCs from normal controls for both the training dataset (AUC = 0.999) and the validation dataset (AUC = 0.983) (Figure 2B). Next, we visualized HCCs and normal controls from the training and validation datasets in two dimensions by principal component analysis (PCA). As expected, HCCs were separated from normal controls, directly demonstrating the accuracy of the diagnostic signature (Figures 2C,D).

### A Potential Biological Indicator for HCC Risk Assessment

Some individuals with primary liver diseases (HBV/HCV viral infection, alcoholism, and cirrhosis) are at high risk for HCC (Marengo et al., 2016; Kennedy and Francis, 2017). To investigate change in methylation levels during the formation of HCC, we calculated the class-score for the diagnostic signature of normal controls, individuals with liver diseases, and HCCs. Class-score was significantly correlated with the risk for HCC. The class-scores of individuals with liver diseases were between the values of those of normal controls and HCCs (Figure 2E). Therefore, the diagnostic signature can be used to evaluate risk for HCC, enabling timely preventive measures to be taken by individuals with a high risk for HCC. Further, the five DNA methylation markers had better performance, better stability, and reliability than other known diagnostic markers (Llovet et al., 2006; Lou et al., 2017; Zheng et al., 2018).

### Constructing and Validating a Prognostic Signature for HCC Based on DNA Methylation

We sampled two-thirds of the HCCs from the TCGA dataset as a training dataset, with the remaining one-third used as a validation dataset (Supplementary Table S4). Select candidate markers were evaluated in the following manner. First, markers with average methylation level greater than 0.2 (Ave_*T*_ >0.2) in the training dataset were selected. Second, to attenuate variable dimensions and enhance the accuracy and interpretability of the prediction model, we prescreened markers with the ability to divide patients into low-risk and high-risk groups. These markers showed a two-fold difference in average survival time for patients with deceased status between the two groups (see section “Materials and Methods”). A total of 647 methylation markers were selected as candidate markers. Third, univariate Cox regression analysis and a LASSO Cox regression model were implemented to further reduce the number of candidate markers. After the above filtering process, 22 methylation markers were retained, and finally incorporated into a multivariate Cox regression model to construct a prognostic signature (Guo et al., 2019) (Supplementary Figure S1). The signature included four of the 22 markers: cg19265480, cg06293745, cg17186803, and cg0815137. The genes corresponding to the four markers were NBPF member 8 (*NBPF8*), ATP binding cassette subfamily B member 1 (*ABCB1*) or RUN domain containing 3B (*RUNDC3B*), sodium voltage-gated channel beta subunit 4 (*SCN4B*), and golgi associated kinase 1A (*GASK1A*), respectively, (Supplementary Table S5, see section “Materials and Methods”). The risk score for HCC based on β-values of the four methylation markers were: risk score = (1.557 × cg19265480) + (4.321 × cg06293745) + (1.556 × cg17186803) – (1.786 × cg08151370). Of these four markers, cg19265480, cg06293745, and cg17186803 had positive coefficients, indicating a correlation between larger DNA methylation β-values and shorter overall survival (OS), while a larger β-value for cg08151370 correlated with longer OS.

**FIGURE 3:**
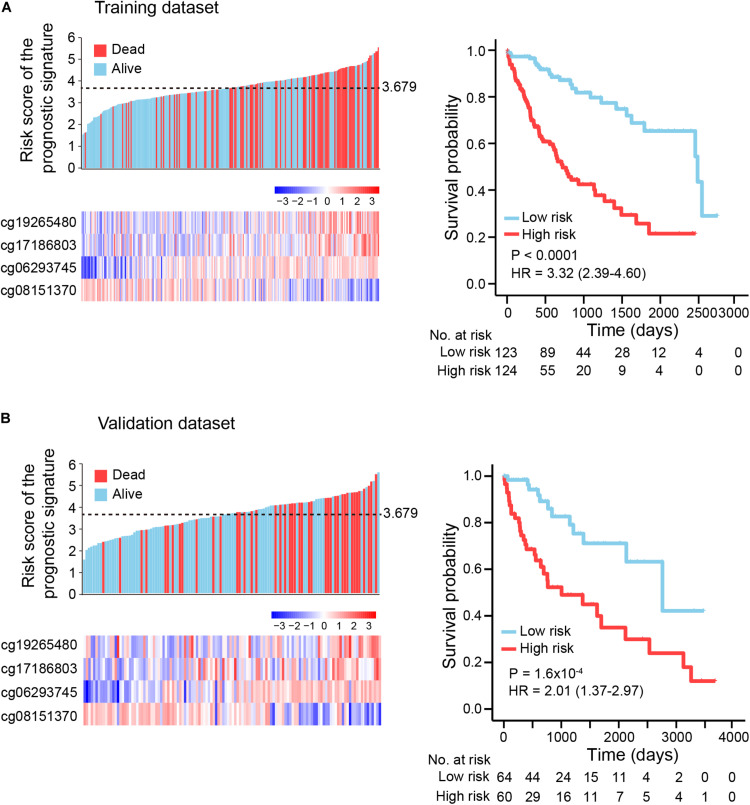
Kaplan-Meier (KM) survival analysis based on the risk score of the prognostic signature. **(A)** Training dataset (two-thirds of TCGA dataset), **(B)** Validation dataset (one-third of TCGA dataset). Upper left panel: the distribution of risk scores and survival status of HCC patients; lower left panel: heatmap showing methylation level of four DNA methylation markers in HCC patients. Right panel: HCC patients were divided into low-risk and high-risk groups using a median cutoff value (black dotted line: 3.679, upper left panel) of risk scores. KM curves along with log-rank test and hazard ratio (HR) were used to compare and visualize the OS of low-risk and high-risk groups.

To explore the association between prognostic signature and OS in HCC in the training and validation datasets, we first assessed the distribution of risk scores and survival status of HCC patients. Patients with lower risk scores (risk-score < 3.679) generally had better survival, as evident from 123 observations with 25 events in the training dataset and 64 observations with 12 events in the validation dataset; patients with higher risk scores (risk-score ≥ 3.679) had relatively poor survival, according to 124 observations with 64 events in the training dataset and 60 observations with 31 events in the validation dataset (Figures 3A,B, left panel). By use of the median risk score (3.679) as the cutoff point, Kaplan-Meier curves were generated for the training and validation datasets. As expected, compared with patients in the high-risk group, patients in the low-risk group had longer OS (training dataset: log-rank test *p* < 1.0E-04, HR: 3.32, 95% CI of HR: 2.39-4.60; validation dataset: log-rank test *p* = 1.6E-04, HR: 2.01, 95% CI of HR: 1.37-2.97) (Figures 3A,B, right panel).

**FIGURE 4:**
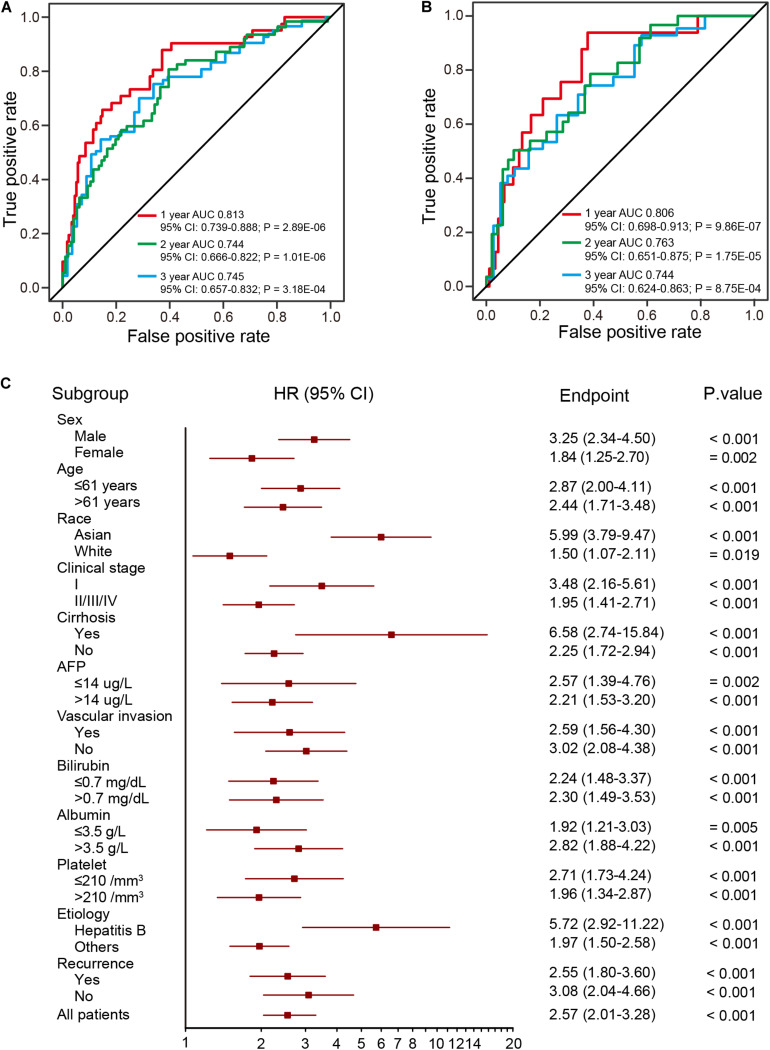
ROC and stratification analysis of HCCs based on the risk score of the prognostic signature. **(A,B)** The ROC analysis of the prognostic signature at three different time points (1 year, 2 years, and 3 years) for the training (two-thirds of TCGA dataset) **(A)** and validation **(B)** datasets (one-third of TCGA dataset). **(C)** HR of overall mortality for TCGA HCC patients using the regroup method and based on different clinical characteristics.

To evaluate the prognostic potential and investigate the specificity and sensitivity of the prognostic signature for survival prediction, AUC values were calculated at 1-year, 2-years, and 3-years by time-dependent ROC analysis. The AUCs estimated for OS were 0.813 at 1 year, 0.744 at 2 years, and 0.745 at 3 years in the training dataset (Figure 4A). For the validation dataset, the AUCs were 0.806 at 1 year, 0.763 at 2 years, and 0.744 at 3 years (Figure 4B). These results indicate the prognostic signature to have great potential as a prognostic biomarker for HCC.

A good prognostic signature should be independent of clinical variables. To assess the independence of the present prognostic signature, survival analysis was performed using subsets of patients with different clinical and pathological characteristics (age, sex, race, AJCC stage, serum AFP levels and etc.), which are known as prevailing predictors (Lo et al., 2002; Njei et al., 2015; Bruix et al., 2016). As shown in Figure 4C, the four-DNA methylation prognostic signature remained statistically and clinically significant as a prognostic model based on these clinical variables. In addition, Kaplan-Meier analysis demonstrated that patients in the high-risk group had significantly (log-rank test, all *p* < 0.05) shorter OS and poorer prognosis, with the AUC values higher than 0.7 (Supplementary Table S6). These results suggest the prognostic signature to be independent of patients’ pathological characteristics.

### Comparison of the Prognostic Signature With Known Prognostic Biomarkers

**FIGURE 5:**
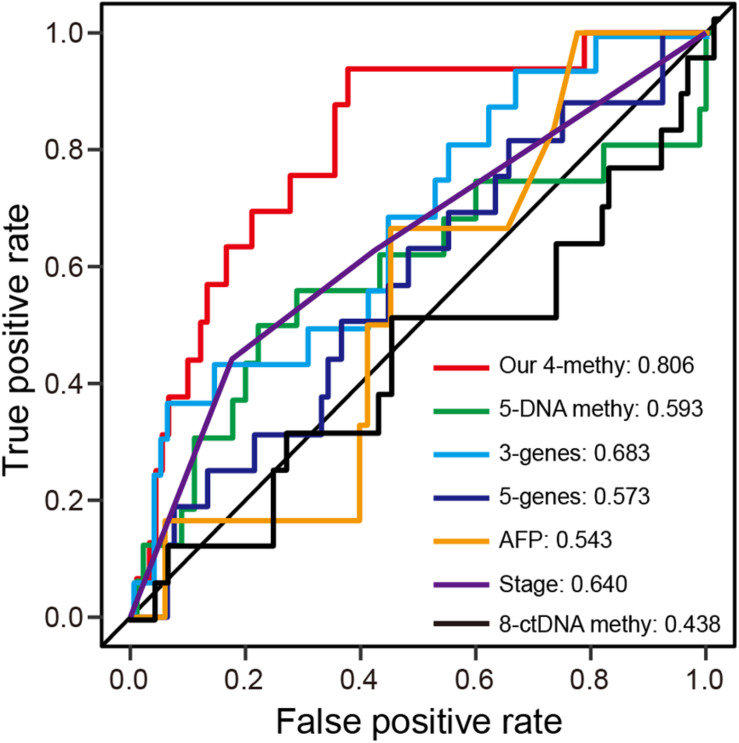
ROC analysis of different prognostic biomarkers. **(A)** ROC curves demonstrate the sensitivity and specificity of the four-DNA methylation marker prognostic signature and other known prognostic biomarkers using the validation dataset (one-third of TCGA dataset).

Numerous prognostic biomarkers have been identified for HCC patients. By comparison of these known biomarkers, we can determine whether the four-DNA methylation prognostic signature in this study shows better performance in terms of predicting patient survival (Xu et al., 2017; Fang et al., 2018; Yang et al., 2018; Li et al., 2019). Using a previously reported formula for calculating risk score, ROC analysis at 1-year was performed with the validation dataset. The results showed the prognostic signature to have the highest AUC value of any of the other known biomarkers (Figure 5A). Hence, the four-DNA methylation prognostic signature had better performance than the other prognostic biomarkers.

### Association of the Prognostic Signature With Gene Expression

DNA methylation plays an important role in regulation of gene expression. Hypermethylation inhibits gene expression while hypomethylation is associated with gene activation (Irizarry et al., 2009; Thienpont et al., 2016; Ma et al., 2017; Wang et al., 2018). Using TCGA transcriptome profiles (Supplementary Table S3), we explored the association between the four-DNA methylation prognostic signature and gene expression. Pearson’s correlations between β-value and gene expression were significantly positive for *NBPF8* (*p* = 8.58E-03) and *ABCB1* (*p* = 1.58E-07), with inverse significance for *FAM198A* (*p* = 5.40E-03) and *SCN4B* (*p* = 0.033). These results demonstrate the four methylation markers of the prognostic signature to be correlated with expression of corresponding genes. To explore the relationship between gene expression and risk score of the prognostic signature and to investigate the possible role of the prognostic signature in HCC therapy, we performed one-to-one correlation analysis between genes of the whole genome and the prognostic signature. The top 10 genes that were significantly positively correlated with the prognostic signature were *KIF2C*, *MYBL2*, *CDC6*, *CDC20*, *CENPA*, *CENPO*, *KIF4A*, *CDCA8*, *KIAA1524*, and *PRR11* (*p* < 0.001). The top 10 genes that were significantly negatively correlated were *PDE2A*, *CYB5D2*, *RAMP3*, *CLEC3B*, *CRY2*, *EMCN*, *DNASE1L3*, *GNA14*, *CSAD*, and *LINC01537* (*p* < 0.001).

### Gene Ontology (GO) Enrichment Analysis

**FIGURE 6:**
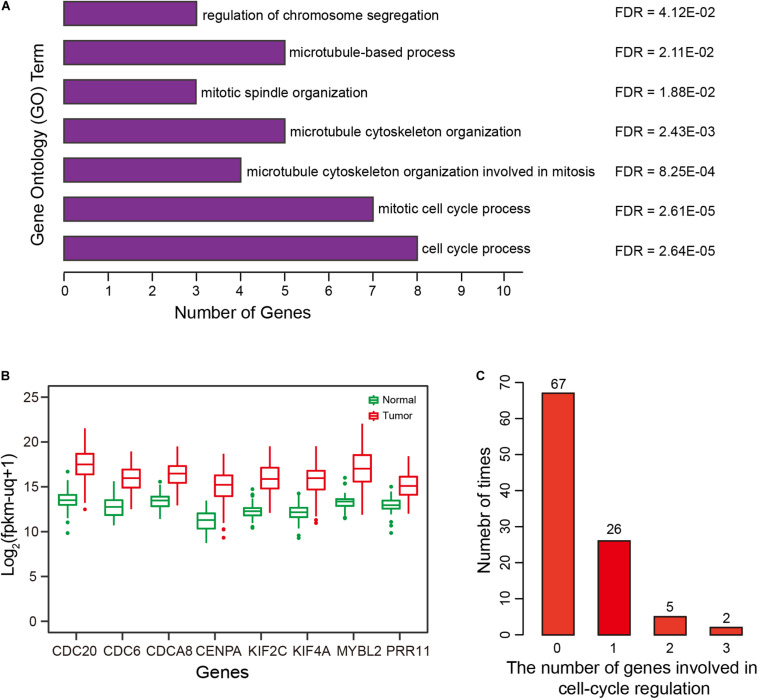
Correlation and Gene Ontology (GO) enrichment analysis. **(A)** GO terms enriched for the top 10 genes significantly and positively correlated with risk scores of the prognostic signature. **(B)** Expression of eight genes by HCCs and normal controls. **(C)** Distribution and number of genes involved in cell cycle regulation, for 100 repetitions.

Biological function underlying the two-correlated gene lists was evaluated by Gene Ontology (GO) functional enrichment analysis using the Gorilla tool (Eden et al., 2009). The top 10 positively correlated genes were mainly involved in “cell cycle process” (GO:0022402), “mitotic cell cycle process” (GO:1903047), “microtubule cytoskeleton organization involved in mitosis” (GO:1902850), and “microtubule cytoskeleton organization” (GO:0000226) (all FDR < 0.01) (Figure 6A). For the top 10 negatively correlated genes, there was no significant GO term enrichment. Intriguingly, eight of top 10 positively correlated genes were involved in the process of cell-cycle regulation (Figure 6A). Of these eight genes, *KIF2C* and *MYBL2* overexpression was associated with shorter OS for HCC patients, therefore, they represent potential therapeutic targets (Chen et al., 2017; Musa et al., 2017). *CDC6* and *CDC20* have been reported to play critical roles in prostate cancer progression, and can serve as indicators for patient prognosis (Karanika et al., 2017; Zhang et al., 2019). *CENPA* combined with *CDK1* and *CDC20* can serve as a cluster of prognostic biomarkers for lung adenocarcinoma (Liu et al., 2018). *KIF4A*, combined with *FOXM1*, can mediate HCC progression (Hu et al., 2019). Some studies have suggested that the *CDCA8-AURKB* pathway is a promising therapeutic target for lung cancer patients via activation of the Wnt/β-catenin signaling pathway (Hayama et al., 2007). *PRR11* plays a critical role in HCC progression (Qiao et al., 2019). As expected, these eight genes were all overexpressed in HCCs (Figure 6B).

### Relationship of Prognostic Signature Genes With Prognosis

To validate the specificity of the results and exclude confounding factors, we randomly selected four methylation markers from among 647 candidate markers, and then performed multivariate-Cox regression analysis to construct a ‘simulated prognostic model (SPM)’. The top 10 genes positively correlated with risk score for SPM were used to perform GO enrichment analysis, and genes related to cell-cycle regulation were counted. We repeated this random process 100 times, results showed that fewer than two genes were related to cell-cycle regulation in 93 of the 100 times, with two or three genes related to cell-cycle regulation for the remaining seven times (Figure 6C). Therefore, compared with random results, the prognostic signature was significantly correlated with cell-cycle regulation (FDR < 0.01).

**FIGURE 7:**
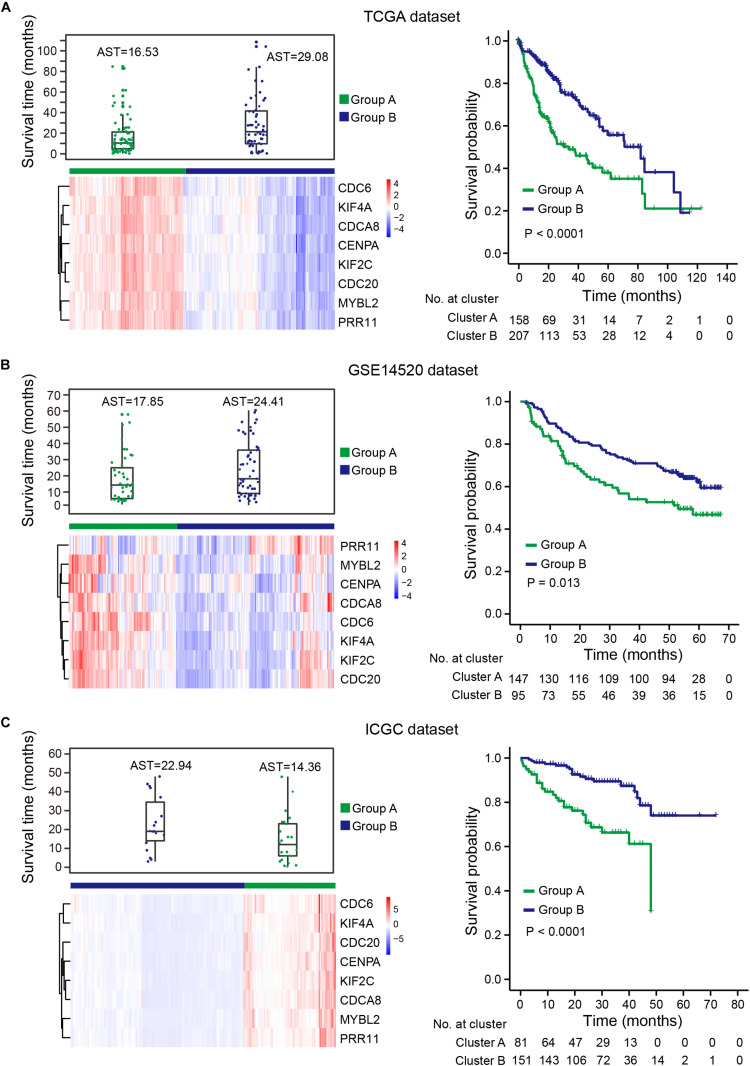
HCCs grouped based on the expression of eight genes. **(A)** TCGA dataset, **(B)** GSE14520 dataset, **(C)** ICGC dataset. Upper left panel: survival time distribution of HCC patients with deceased status in group A (green) and group B (blue). Lower left panel: unsupervised hierarchical clustering of eight genes for HCC patients, patients were divided into two groups based on the expression of the eight genes. Right panel: KM survival analysis for HCC patients in the two groups. Group B had a better prognosis and OS than group A. AST: average survival time.

Uncontrolled cell proliferation arising from aberrant activity of various cell cycle proteins is an obvious feature of tumors, with cell cycle proteins considered to be promising targets for tumor therapy (Dominguez-Brauer et al., 2015; Otto and Sicinski, 2017). Thus, the group of eight genes that was significantly correlated with cell cycle regulation should have the potential to predict prognosis in HCC. To verify this hypothesis, gene expression profiles were obtained from TCGA, ICGC-LIRI-JP, and GSE14520 (Supplementary Table S3). Unsupervised hierarchical clustering of the eight genes showed that HCCs were successfully divided into two groups, with the average survival time of patients with deceased status in the low-expression group greater than patients with deceased status in the high-expression group (Figures 7A–C, left panel). Kaplan-Meier curves along with the log-rank test were used to compare and visualize the OS of patients in the two groups. As expected, the survival of patients in the eight-gene low-expression group was significantly improved in comparison with that in patients in the other group (Figures 7A–C, right panel). These results indicate the eight genes to potentially serve as prognostic biomarkers. These eight genes are highly correlated with the prognostic signature, which provides indirect evidence to demonstrate the validity of the prognostic signature.

## Discussion

With the development of whole-genome technologies, multiple diagnostic and prognostic signatures have been established using methylation markers, mRNA, microRNA, and single nucleotide polymorphism (SNP) datasets (Liu et al., 2012; Yoon et al., 2014; Fang et al., 2018; Li et al., 2019). However, previous studies in this area have several obvious limitations: (1) They typically focused on patients with a specific clinical feature, or a single gene or methylation marker; (2) Too many markers were integrated into one signature, which reduced the potential for clinical use; (3) The design of the prediction signature failed to provide a quantitative risk score of death for each patient, and the formula for risk score was unavailable; (4) Signatures were not validated in multiple independent datasets; (5) The biological mechanism underlying the signature were unclear. These shortcomings prevent the use of signatures in clinical practice. In this study, multiple methylation and transcriptome profiles from different sources were used to construct and validate diagnostic and prognostic signatures. The diagnostic and prognostic signatures consisted of five DNA methylation markers and four DNA methylation markers, respectively.

Alterations in DNA methylation are a molecular hallmark of tumors, and are the most intensively studied epigenetic phenomena. Disturbances to methylation levels can alter gene transcription, thus modifying the biological behavior of tumors (Irizarry et al., 2009; Hattori and Ushijima, 2016; Thienpont et al., 2016; Ma et al., 2017; Koch et al., 2018; Wang et al., 2018). To date, multiple studies have shown that DNA methylation is directly involved in carcinogenesis, with altered DNA methylation often observed prior to actual tumor formation (Issa et al., 2001; Brait et al., 2009; Shenoy et al., 2015). A similar phenomenon was found for our diagnostic signature: the class-scores had an upward trend in individuals with liver diseases compared with that in normal controls. Class-scores were significantly different between normal controls, individuals with liver diseases, and HCCs. Hence, this diagnostic signature can be used to monitor the health status of individuals.

In previous studies, circulating tumor DNA (ctDNA) methylation markers were used to construct prognostic model (Xu et al., 2017). However, ctDNA is likely released by apoptotic cells and may therefore represent a particular subtype of tumor cells, with differences in methylation levels between ctDNA and tissue DNA (Diaz and Bardelli, 2014). Therefore, ctDNA methylation markers are not as suitable as tissue DNA for diagnostic and prognostic purposes, with ctDNA performing less well in a prognostic prediction model than other known biomarkers (Figure 5A). Prognostic signatures can predict recurrence for early stage HCC (Qiu et al., 2017). The use of methylation array analysis enabled stratification of HCCs into different CpG island methylation phenotypic subtypes that can be used to build prognostic models based on differential subtype genes (Li et al., 2019). To date, a comprehensive and systemic approach to build prognostic signatures based on methylation markers remains unavailable. Using the TCGA methylation dataset, we successfully built a prognostic signature independent of clinical variables. Because epigenetic changes can alter gene expression, there is a causal relationship between methylation level and gene expression (Irizarry et al., 2009; Thienpont et al., 2016; Ma et al., 2017; Wang et al., 2018). Intriguingly, correlation analysis between methylation and gene expression showed that eight of the top 10 positively correlated genes were involved in regulation of cell cycle progression. These eight genes performed as well as our prognostic signature for analysis of progression. These findings demonstrate the superior performance of our prognostic signature in terms of predicting HCC OS. We will further validate this prognostic signature when new clinical samples are available.

In conclusion, based on multiple independent datasets from different sources, we constructed an accurate five-DNA methylation diagnostic signature with excellent specificity and sensitivity, which was additionally cost-effective for clinical application. Further, we identified and verified a four-DNA methylation prognostic signature, which was not only independent of clinical factors, but also had better performance for OS prediction than other known biomarkers. Analysis of transcriptome profiles revealed that eight of the top 10 genes positively correlated with risk score for HCCs were primarily involved in cell cycle regulation. This eight-gene panel is a potential prognostic biomarker.

## Data Availability Statement

Publicly available datasets were analyzed in this study, these can be found in The Cancer Genome Atlas via (https://xenabrowser.net/datapages/); the NCBI Gene Expression Omnibus and the International Cancer Genomics Consortium (ICGC, LIRI-JP).

## Ethics Statement

The animal study was reviewed and approved by the Ethics Committees of participating hospitals.

## Author Contributions

RL and LS performed the data analysis and wrote the analysis pipeline. CW wrote the manuscript. JJ provided part of the analysis methods in diagnostic signature. All authors read and approved the final manuscript.

## Conflict of Interest

JJ was employed by company Zhongtong-Lanbo Diagnostic LTD, China. The remaining authors declare that the research was conducted in the absence of any commercial or financial relationships that could be construed as a potential conflict of interest.

## Footnotes

1 https://xenabrowser.net/datapages/

2 https://gdc.xenahubs.net/download

## Supplementary Material

FIGURE S1 Workflow for data analysis. **(A)** Left panel: construction of the diagnostic signature: we identified differential methylation markers in the TCGA training dataset and ranked candidate markers by balanced accuracy. We constructed an optimal five-DNA methylation marker diagnostic signature with an increase in methylation markers. The diagnostic signature was validated in a mixed dataset consisting of six independent datasets. Right panel: construction of the prognostic signature: we first selected candidate markers that have potential to distinguish high-risk and low-risk HCC patients. Then univariate Cox, LASSO Cox, and multivariate Cox model were used to construct a four-DNA methylation marker prognostic signature. The function of genes significantly correlated with risk scores of HCC patients was also analyzed with three independent datasets.

FIGURE S2 Process for construction of the diagnostic signature. **(A)** With an increase in methylation markers, we constructed a diagnostic signature with the highest average balanced accuracy using multiple simulations. BA: balanced accuracy. **(B)** The curve of average balanced accuracy with increased numbers of methylation markers at 10, 50, 300, 500, and 1000 simulated assessments. Right panel: the red dotted line indicates the diagnostic signature with five DNA methylation markers to have the highest balanced accuracy after 300 simulations.

TABLE S1 The result of the simulation.

TABLE S2 Characteristics of five methylation markers used in the diagnostic signature.

TABLE S3 Information regarding datasets used in this study.

TABLE S4 Clinical characteristics of HCC patients in the TCGA dataset.

TABLE S5 Characteristics of four methylation markers used in the prognostic signature.

TABLE S6 Results of KM and ROC analysis based on different regrouping methods.
