# Supplementary figures and images for: Construction and Validation of Novel Diagnostic and Prognostic DNA Methylation Signatures for Hepatocellular Carcinoma
Source: Front Genet. 2020 Aug 13;11:906. doi: 10.3389/fgene.2020.00906 (PMC7456968; doi:10.3389/fgene.2020.00906)

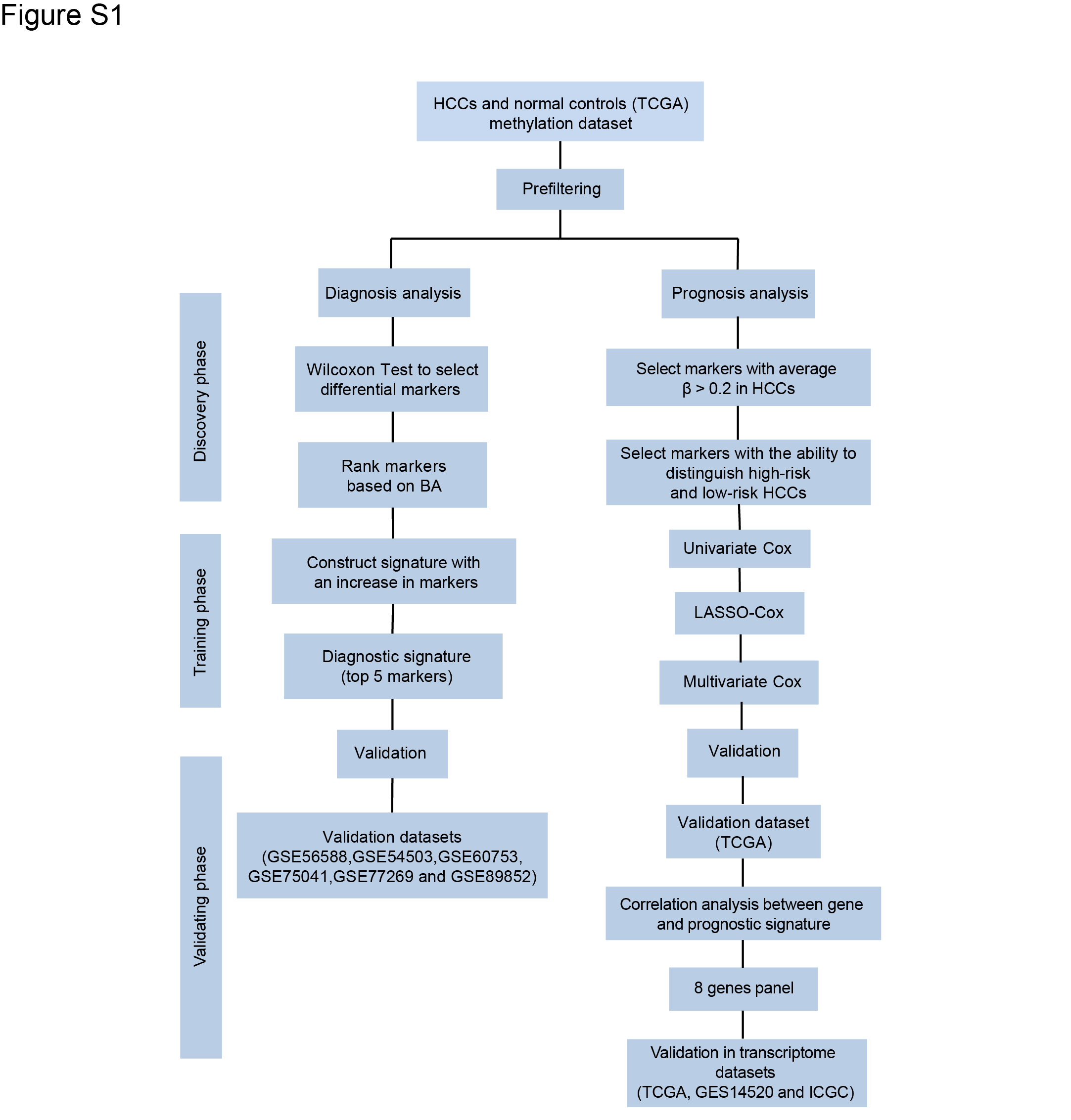

Supplement: FIGURE S1 — Workflow for data analysis. (A) Left panel: construction of the diagnostic signature: we identified differential methylation markers in the TCGA training dataset and ranked candidate markers by balanced accuracy. We constructed an optimal five-DNA methylation marker diagnostic signature with an increase in methylation markers. The diagnostic signature was validated in a mixed dataset consisting of six independent datasets. Right panel: construction of the prognostic signature: we first selected candidate markers that have potential to distinguish high-risk and low-risk HCC patients. Then univariate Cox, LASSO Cox, and multivariate Cox model were used to construct a four-DNA methylation marker prognostic signature. The function of genes significantly correlated with risk scores of HCC patients was also analyzed with three independent datasets. [file Image_1.TIF]

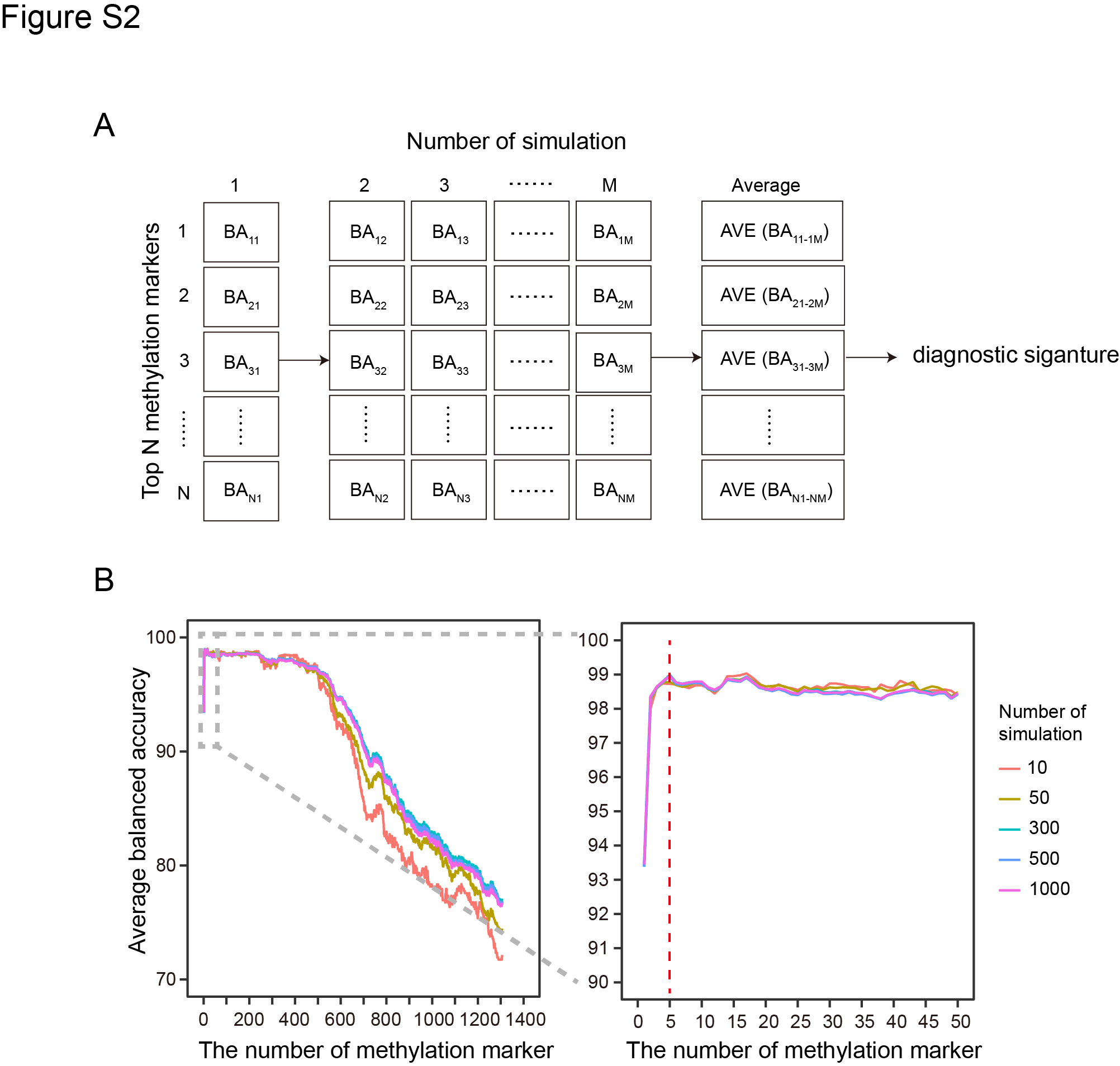

Supplement: FIGURE S2 — Process for construction of the diagnostic signature. (A) With an increase in methylation markers, we constructed a diagnostic signature with the highest average balanced accuracy using multiple simulations. BA: balanced accuracy. (B) The curve of average balanced accuracy with increased numbers of methylation markers at 10, 50, 300, 500, and 1000 simulated assessments. Right panel: the red dotted line indicates the diagnostic signature with five DNA methylation markers to have the highest balanced accuracy after 300 simulations. [file Image_2.TIF]
