# Supplementary material for: Construction and Validation of Novel Diagnostic and Prognostic DNA Methylation Signatures for Hepatocellular Carcinoma
Source: Front Genet. 2020 Aug 13;11:906. doi: 10.3389/fgene.2020.00906 (PMC7456968; doi:10.3389/fgene.2020.00906)
Supplement: TABLE S2 — Characteristics of five methylation markers used in the diagnostic signature. [file Table_2.DOCX]

**Supplementary Table 2.** Characteristics of five methylation markers used in the diagnostic signature

| **Probe ID** | **Chromosomal location** | **Gene symbol** | **CGI coordinate** | **Feature type** | **AML (N)** | **AML (T)** | **Log2FC** | **P.adjust** |
| --- | --- | --- | --- | --- | --- | --- | --- | --- |
| cg24985525 | chr11:68351263-68351264 | *LRP5* | chr11:68347849-68348258 | S_Shelf | 0.407 | 0.891 | 1.131 | 3.05E-23 |
| cg24035245 | chr1:118993305-118993306 | *TBX15* | chr1:118993043-118993364 | Island | 0.235 | 0.698 | 1.574 | 8.08E-23 |
| cg21072795 | chr12:54527147-54527148 | *NCKAP1L* | chr12:54588149-54588807 | NA | 0.427 | 0.146 | -1.553 | 4.52E-23 |
| cg07274716 | chr5:135028283-135028284 | *PITX1* | chr5:135027402-135029456 | Island | 0.206 | 0.646 | 1.650 | 1.00E-22 |
| cg14188840 | chr7:27180779-271807  80 | *HOXA10* | chr7:27179690-27180131 | S_Shore | 0.402 | 0.805 | 1.002 | 1.14E22 |

AML(N): average methylation level in normal patients

AML(T): average methylation level in tumor patients

P.adjust: adjusted P value with multiple testing-corrected Wilcoxon test
