# Supplementary material for: Construction and Validation of Novel Diagnostic and Prognostic DNA Methylation Signatures for Hepatocellular Carcinoma
Source: Front Genet. 2020 Aug 13;11:906. doi: 10.3389/fgene.2020.00906 (PMC7456968; doi:10.3389/fgene.2020.00906)
Supplement: TABLE S3 — Information regarding datasets used in this study. [file Table_3.DOCX]

| **Data set** | **Methylation dataset** | | | **Gene expression dataset** | | |
| --- | --- | --- | --- | --- | --- | --- |
|  | **Source** | **#T** | **#NT** | **Source** | **#T** | **#NT** |
| DS1 | TCGA | 371 | 50 | TCGA | 371 | 50 |
| DS2 | GSE54503 | 66 | 66 | ICGC (LIRI-JP) | 232 |  |
| DS3 | GSE56588 | 224 | 19 | GSE14520 | 242 |  |
| DS4 | GSE60753 | 32 | 111 |  |  |  |
| DS5 | GSE75041 | 66 | 0 |  |  |  |
| DS6 | GSE77269 | 40 | 20 |  |  |  |
| DS7 | GSE89852 | 37 | 37 |  |  |  |
| Totals |  | 836 | 303 |  | 845 | 50 |

**Supplementary Table 3.** Information regarding datasets used in this study

#T: The number of tumor samples

#NT: The number of the non-tumor samples
