# Supplementary material for: Construction and Validation of Novel Diagnostic and Prognostic DNA Methylation Signatures for Hepatocellular Carcinoma
Source: Front Genet. 2020 Aug 13;11:906. doi: 10.3389/fgene.2020.00906 (PMC7456968; doi:10.3389/fgene.2020.00906)
Supplement: TABLE S4 — Clinical characteristics of HCC patients in the TCGA dataset. [file Table_4.DOCX]

**Supplementary Table 4.** Clinical characteristics of HCC patients in the TCGA dataset

|  |  | **Patients** | | | | | |
| --- | --- | --- | --- | --- | --- | --- | --- |
|  |  | **Total (N=371)** | | **Training (N=247)** | | **Validation (N=124)** | |
| **Characteristics** | **Groups** | **No** | **%** | **No** | **%** | **No** | **%** |
| Sex | Male | 251 | 67.65 | 168 | 68.02 | 83 | 66.94 |
|  | Female | 120 | 32.35 | 79 | 31.98 | 41 | 33.06 |
| Age at diagnosis | Median | 61.0 | | 61.0 | | 61.0 | |
|  | ≤61 | 191 | 51.48 | 127 | 51.42 | 60 | 48.39 |
|  | >61 | 180 | 48.52 | 120 | 48.58 | 64 | 51.61 |
| Pathologic stage | I | 174 | 46.90 | 110 | 44.53 | 64 | 51.61 |
|  | II | 85 | 22.91 | 55 | 22.27 | 30 | 24.19 |
|  | III | 84 | 22.64 | 60 | 24.29 | 24 | 19.35 |
|  | IV | 4 | 1.08 | 3 | 1.21 | 1 | 0.81 |
|  | Others | 24 | 6.47 | 19 | 7.69 | 5 | 4.03 |
| Race | Asian | 158 | 42.59 | 108 | 43.72 | 50 | 40.32 |
|  | White | 185 | 49.87 | 122 | 49.39 | 63 | 50.81 |
|  | Others | 28 | 371 | 17 | 6.88 | 11 | 8.87 |
| Fibrosis | Cirrhosis | 79 | 21.29 | 53 | 21.46 | 26 | 20.97 |
|  | Fibrosis | 60 | 16.17 | 30 | 12.15 | 30 | 24.19 |
|  | No fibrosis | 76 | 20.49 | 53 | 21.46 | 23 | 18.55 |
|  | Others | 156 | 42.05 | 111 | 44.94 | 45 | 36.29 |
| Recurrence | YES | 172 | 46.36 | 115 | 46.56 | 57 | 45.97 |
|  | NO | 181 | 48.79 | 120 | 48.58 | 61 | 49.19 |
|  | Others | 18 | 4.85 | 12 | 4.86 | 6 | 4.84 |
| Vascular invasion | Micro/Macro | 108 | 29.11 | 78 | 31.58 | 30 | 24.19 |
|  | None | 209 | 56.33 | 132 | 53.44 | 77 | 62.10 |
|  | Others | 54 | 14.56 | 37 | 14.98 | 17 | 13.71 |
| AFP(ug/L) | ≤14 | 142 | 38.27 | 99 | 40.08 | 43 | 34.68 |
|  | >14 | 140 | 37.74 | 93 | 37.65 | 47 | 37.90 |
|  | Others | 89 | 23.99 | 55 | 22.27 | 34 | 27.42 |
| Vital Status | Alive | 239 | 64.42 | 158 | 63.97 | 81 | 65.32 |
|  | Dead | 132 | 35.58 | 89 | 36.03 | 43 | 34.68 |
| Etiology | Hepatitis C | 49 | 13.21 | 29 | 11.74 | 20 | 16.13 |
|  | Hepatitis B | 98 | 26.42 | 64 | 25.91 | 34 | 27.42 |
|  | Alcohol | 77 | 20.75 | 55 | 22.27 | 22 | 17.74 |
|  | Others | 147 | 39.62 | 99 | 40.08 | 48 | 38.71 |
| Bilirubin (mg/dL) | ≤0.7 | 173 | 46.63 | 114 | 46.15 | 59 | 47.58 |
|  | >0.7 | 133 | 35.85 | 90 | 36.44 | 43 | 34.68 |
| Albumin(g/L) | ≤4 | 159 | 42.86 | 113 | 45.75 | 46 | 37.10 |
|  | >4 | 143 | 38.54 | 88 | 35.63 | 55 | 44.35 |
| Platelet count (/mm^3^) | ≤210 | 155 | 41.78 | 98 | 39.68 | 57 | 45.97 |
|  | >210 | 154 | 41.51 | 106 | 42.91 | 48 | 38.71 |
