# Supplementary material for: Construction and Validation of Novel Diagnostic and Prognostic DNA Methylation Signatures for Hepatocellular Carcinoma
Source: Front Genet. 2020 Aug 13;11:906. doi: 10.3389/fgene.2020.00906 (PMC7456968; doi:10.3389/fgene.2020.00906)
Supplement: TABLE S5 — Characteristics of four methylation markers used in the prognostic signature. [file Table_5.DOCX]

**Supplementary Table 5.** Characteristics of four methylation markers used in the prognostic signature

| **Probe ID** | **Chromosomal location** | **Gene symbol** | **CGI coordinate** | **Feature type** | **AML (T)** | ***P* value^a^** | **Coef.^b^** | ***P* value^b^** |
| --- | --- | --- | --- | --- | --- | --- | --- | --- |
| cg19265480 | chr1:148309779-148309  780 | *NBPF8* | chr1:148309958-14831  0365 | N_Shore | 0.450 | 3.56E-04 | 1.557 | 2.56E-02 |
| cg06293745 | chr7:87700091-877000  92 | *ABCB1; RUNDC3B* | chr7:87627642-87629  128 | NA | 0.813 | 8.26E-03 | 4.321 | 2.77E-03 |
| cg17186803 | chr11:118152323-118152  324 | *SCN4B* | chr11:118152637-11815  3479 | N_Shore | 0.384 | 2.76E-06 | 1.556 | 4.02E-02 |
| cg08151370 | chr3:43031660-430316  61 | *FAM198A* | chr3:43079929-43080  177 | NA | 0.621 | 9.50E-05 | -1.786 | 4.72E-03 |

AML(T): average methylation level in tumor patients

^a^. in univariate Cox regression analysis;

^b^. in multivariate Cox regression analysis;
