# Supplementary material for: Construction and Validation of Novel Diagnostic and Prognostic DNA Methylation Signatures for Hepatocellular Carcinoma
Source: Front Genet. 2020 Aug 13;11:906. doi: 10.3389/fgene.2020.00906 (PMC7456968; doi:10.3389/fgene.2020.00906)
Supplement: TABLE S6 — Results of KM and ROC analysis based on different regrouping methods. [file Table_6.DOCX]

| **Regrouping factors** | **Group** | **Sample size** | **Kaplan-Meier, P value** | **AUC** | **95% CI of AUC** |
| --- | --- | --- | --- | --- | --- |
| Sex | Male | 251 | 2.42E-12 | 0.84 | 0.78-0.91 |
|  | Female | 120 | 1.01E-02 | 0.76 | 0.63-0.89 |
| Age | ≤61 | 191 | 2.74E-06 | 0.83 | 0.74-0.93 |
|  | >61 | 180 | 7.42E-08 | 0.78 | 0.70-0.87 |
| Race | Asian | 158 | 2.07E-13 | 0.88 | 0.82-0.95 |
|  | White | 185 | 1.55E-02 | 0.77 | 0.66-0.88 |
| Clinical stage | I | 174 | 9.14E-08 | 0.82 | 0.72-0.92 |
|  | II/III/IV | 173 | 1.86E-04 | 0.78 | 0.69-0.86 |
| Cirrhosis | Yes | 70 | 1.21E-05 | 0.88 | 0.78-0.99 |
|  | No | 301 | 2.40E-08 | 0.80 | 0.73-0.87 |
| AFP (ug/L) | ≤14 | 142 | 3.30E-03 | 0.91 | 0.85-0.98 |
|  | >14 | 140 | 5.07E-05 | 0.71 | 0.58-0.83 |
| Vascular invasion | Yes | 108 | 2.37E-03 | 0.75 | 0.61-0.89 |
|  | No | 209 | 1.59E-07 | 0.83 | 0.73-0.92 |
| Bilirubin (mg/dL) | ≤0.7 | 173 | 5.28E-03 | 0.75 | 0.58-0.92 |
|  | >0.7 | 133 | 2.84E-06 | 0.78 | 0.66-0.90 |
| Albumin (g/L) | ≤3.5 | 86 | 3.56E-03 | 0.74 | 0.62-0.87 |
|  | >3.5 | 216 | 2.80E-05 | 0.78 | 0.64-0.93 |
| Platelet (/mm^3^) | ≤210 | 155 | 1.35E-06 | 0.80 | 0.70-0.91 |
|  | >210 | 154 | 5.18E-03 | 0.77 | 0.63-0.91 |
| Etiology | Hepatitis B | 98 | 3.46E-07 | 0.87 | 0.75-0.99 |
|  | Others | 273 | 2.96E-06 | 0.79 | 0.72-0.86 |
| Recurrence | Yes | 172 | 1.04E-05 | 0.84 | 0.77-0.91 |
|  | No | 181 | 3.80E-08 | 0.79 | 0.70-0.88 |

**Supplementary Table 6**. Results of Kaplan-Meier and ROC analysis based on different regrouping methods
